# Supplementary material for: West Nile Virus Seroprevalence in Wild Birds and Equines in Madrid Province, Spain
Source: Vet Sci. 2024 Jun 7;11(6):259. doi: 10.3390/vetsci11060259 (PMC11209238; doi:10.3390/vetsci11060259)
Supplement: Supplementary file 1 [file vetsci-11-00259-s001.zip › vetsci-3004194-supplementary.pdf]

Table S1. West Nile virus (WNV) detection records recovered from class Aves, using PCR and sequencing (PCR), virus-neutralization test (VNT) or competitive enzyme-linked immunosorbent assay (cELISA). WNV, or WN antibodies, has been detected from at least 69 bird species, from 29 families and 16 orders in Spain. Detections have occurred in the following autonomous communities: Andalusia (A), Canary Islands (CI), Castilla La Mancha (CLM), Catalonia (C) and Extremadura (E).

Class	Family	Species	Common name	Autonomous Community	Method	Reference
Accipitriformes	Accipitridae	*Accipiter gentilis*	Goshawk	A, C	PCR, VNT	[18,26,66]
		Accipiter nisus	Sparrowhawk	C, E	cELISA, VNT	[39,67]
		Aegypius monachus	Black vulture	A, E	cELISA, VNT	[39,71]
		Aquila adalberti	Spanish imperial eagle	A	cELISA, VNT	[71]
		Aquila chrysaetos	Golden eagle	A, CLM, E	cELISA, PCR, VNT	[16,67]
		Aquila fasciatus	Bonelli's eagle	A, CLM, C	cELISA, VNT, PCR	[16,32,71]
		Buteo buteo	Buzzard	C, E	cELISA, VNT	[39,32]
		Circaetus gallicus	Short-toed snake eagle	A, C, E	cELISA, VNT	[16,39,67]
		Circus aeruginosus	Marsh harrier	E	cELISA, VNT	[39]
		Circus cyaneus	Hen harrier	C	cELISA, VNT	[67]
		Circus pygargus	Montagu's harrier	A, E	cELISA, VNT	[16,30,39,71]
		Gypaetus barbatus	Bearded vulture	C	VNT	[32]
		Gyps fulvus	Griffon vulture	A	cELISA, VNT	[72]
		Hieraaetus pennatus	Booted eagle	A	cELISA, VNT	[30,72,73]
		Milvus migrans	Black kite	A, C, E	cELISA, VNT	[39,67,71,72]
		Milvus milvus	Red kite	C, E	cELISA, VNT	[32,39,67]
		Neophron percnopterus	Egyptian vulture	A	cELISA	[71]
		Pernis apivorus	Honey buzzard	C, E	cELISA, VNT	[32,39,67]
Anseriformes	Anatidae	*Anas platyrhynchos*	Mallard	A	cELISA, VNT	[18,30]
		Anser anser	Greylag geese	A	cELISA, VNT	[30]
		Chen canagica	Emperor goose	A	cELISA, VNT	[68]
		Marmoronetta angustirostris	Marbled duck	A	VNT	[18]
Bucerotiformes	Upupidae	*Upupa epops*	Hoopoe	A	cELISA, VNT	[47]
Caprimulgiformes	Caprimulgidae	*Caprimulgus ruficollis*	Red-necked nightjar	C	cELISA, VNT	[67]
Charadriiformes	Laridae	*Larus fuscus*	Lesser black-backed gull	A	cELISA, VNT	[72]

		Larus michahellis	Yellow-legged gull	A, C	cELISA, VNT	[67,69]
Ciconiformes	Ciconiidae	*Ciconia ciconia*	White stork	A, C, E	cELISA, VNT	[18,32,39,67,68]
		Ciconia nigra	Black stork	E	cELISA, VNT	[39]
Columbiformes	Columbidae	*Columba palumbus*	Wood pigeon	C	VNT	[32]
		Columbia livia	Rock dove	A, C	cELISA, VNT	[32,68]
Falconiformes	Falconidae	*Falco eleonorae*	Eleanora's falcon	CI	VNT	[70]
		Falco naumanni	Lesser kestrel	A	VNT	[18]
		Falco subbuteo	Eurasian hobby	C	cELISA, VNT	[39]
		Falco tinnunculus	Kestrel	A, C, E	cELISA, VNT	[18,32,39,67]
Galliformes	Phasianidae	*Alectoris rufa*	Red-legged partridge	A, C	cELISA, VNT	[19,32]
		Gallus gallus	Domestic fowl	C	VNT	[32]
		Phasianus colchicus	Pheasant	A	cELISA, VNT	[19]
Gruiformes	Rallidae	*Fulica atra*	Common coot	A	cELISA, VNT	[11,18,71]
Otidiformes	Otididae	*Otis tarda*	Great bustard	A, E	cELISA	[39,71]
Passeriformes	Acrocephalidae	*Acrocephalus scirpaceus*	Eurasian reed warbler	A	cELISA, VNT	[47]
	Cettidae	*Cettia cetti*	Cetti's warbler	E	cELISA, VNT	[66]
	Corvidae	*Corvus corax*	Common raven	C, E	cELISA, VNT	[32,39]
		Cyanopica cyanus	Azure-winged magpie	A	cELISA, VNT	[47]
		Pica pica	Common magpie	CLM, C	cELISA, PCR, VNT	[16,32,75]
	Estrildidae	*Amandava amandava*	Red avadavat	E	cELISA, VNT	[17]
		Estrilda astrild	Common waxbill	E	cELISA	[17]
	Hirundidae	*Delichon urbicum*	House martin	E	cELISA, VNT	[17,39]
	Lanidae	*Lanius senator*	Woodchat shrike	A	VNT	[73]
	Motacillidae	*Motacilla flava*	Western yellow wagtail	A	cELISA, VNT	[47]
	Muscicapidae	*Erithracus rubecula*	European robin	E	cELISA	[66]
		Phoenicurus ochrurus	Black redstart	A	VNT	[73]
		Phoenicurus phoenicurus	Common redstart	A	cELISA, VNT	[47]
	Passeridae	*Passer domesticus*	House sparrow	A, E	cELISA, VNT	[17,74]
	Ploceidae	*Euplectes afer*	Yellow-crowned bishop	E	cELISA	[17]
	Sylviidae	*Sylvia borin*	Garden warbler	A	VNT	[73
		Sylvia melanocephala	Sardinian warbler	A, E	cELISA, VNT	[17,47
	Turdidae	*Turdus merula*	Blackbird	A	cELISA, VNT	[47,73]
Pelecaniformes	Ardeidae	*Ardea cinerea*	Grey heron	C, E	cELISA, VNT	[39,67]
		Bubulcus ibis	Western cattle egret	A	VNT	[18]

		Egretta garzetta	Little egret	C	cELISA	[67]
	Threskiornithidae	*Platalea leucorodia*	Spoonbill	A	cELISA	[71]
		Plegadis falcinellus	Glossy ibis	A	VNT	[69]
Phoenicopteriformes	Phoenicopteridae	*Phoenicopterus ruber*	Greater flamingo	A	VNT	[69]
Strigiformes	Strigidae	*Asio otus*	Long-eared owl	A, C	cELISA, VNT	[30,67]
		Bubo bubo	Eagle owl	A, E	cELISA, VNT	[18,30,39,72]
		Otus scops	Eurasian scops owl	A, C	cELISA, VNT	[30,39]
		Strix aluco	Tawny owl	A, C, E	cELISA, VNT	[39,18,67,72]
	Tytonidae	*Tyto alba*	Barn owl	A, C, E	cELISA, VNT	[39,67,72]
Struthiorniformes	Struthionidae	*Struthio camelus*	Ostrich	A	cELISA	[68]

NOTE: This list represents a fraction of the available literature, suggesting that the actual number of relevant studies is higher.

References

11. Figuerola, J.; Soriguer, R.; Rojo, G.; Tejedor, C.G.; Jimenez-Clavero, M.A. Seroconversion in wild birds and local circulation of West Nile virus, Spain. *Emerg Inf Dis* **2007**, *13*, 1915-1917.

16. Jimenez-Clavero, M.A.; Sotelo, E.; Fernandez-Pinero, J.; Llorente, F.; Blanco, J.M.; Rodriguez-Ramos, J.; Perez-Ramirez, E.; Hofle, U. West Nile virus in golden eagles, Spain, 2007. *Emerg Infect Dis* **2008**, *14*, 1489-1491.

17. Marzal, A.; Ferraguti, M.; Muriel, J.; Magallanes, S.; Ortiz, J.A.; Garcia-Longoria, L.; Bravo-Barriga, D.; Guerrero-Carvajal, F.; Aguilera-Sepulveda, P.; Llorente, F.; et al. Circulation of zoonotic flaviviruses in wild passerine birds in Western Spain. *Vet. Microbiol.* **2022**, *268*, 5.

18. Lopez, G.; Jimenez-Clavero, M.A.; Vazquez, A.; Soriguer, R.; Gomez-Tejedor, C.; Tenorio, A.; Figuerola, J. Incidence of West Nile virus in birds arriving in wildlife rehabilitation centers in southern Spain. *Vector Borne Zoonotic Dis* **2011**, *11*, 285-290.

19. Llorente, F.; Pérez-Ramírez, E.; Fernández-Pinero, J.; Soriguer, R.; Figuerola, J.; Jiménez-Clavero, M.A. Flaviviruses in game birds, southern Spain, 2011-2012. *Emerg Infect Dis* **2013**, *19*, 1023-1025.

26. Aguilera-Sepulveda, P.; Napp, S.; Llorente, F.; Solano-Manrique, C.; Molina-Lopez, R.; Obon, E.; Sole, A.; Jimenez-Clavero, M.A.; Fernandez-Pinero, J.; Busquets, N. West Nile virus lineage 2 spreads westwards in Europe and overwinters in North-Eastern Spain (2017-2020). *Viruses* **2022**, *14*, Article 569.

30. Jurado-Tarifa, E.; Napp, S.; Lecollinet, S.; Arenas, A.; Beck, C.; Cerda-Cuellar, M.; Fernandez-Morente, M.; Garcia-Bocanegra, I. Monitoring of West Nile virus, Usutu virus and Meaban virus in waterfowl used as decoys and wild raptors in southern Spain. *Comp Immunol* **2016**, *49*, 58-64.

32. Napp, S.; Llorente, F.; Beck, C.; Jose-Cunilleras, E.; Soler, M.; Pailler-Garcia, L.; Amaral, R.; Aguilera-Sepulveda, P.; Pifarre, M.; Molina-Lopez, R.; et al. Widespread circulation of Flaviviruses in horses and birds in northeastern Spain (Catalonia) between 2010 and 2019. *Viruses* **2021**, *13*, Article 2404.

39. Bravo-Barriga, D.; Aguilera-Sepulveda, P.; Guerrero-Carvajal, F.; Llorente, F.; Reina, D.; Perez-Martin, J.E.; Jimenez-Clavero, M.A.; Frontera, E. West Nile and Usutu virus infections in wild birds admitted to rehabilitation centres in Extremadura, western Spain, 2017-2019. *Vet Microbiol* **2021**, *255*, Article 10.
47. Ferraguti, M.; Martinez-De la Puente, J.; Soriguer, R.; Llorente, F.; Jimenez-Clavero, M.A.; Figuerola, J. West Nile virus-neutralizing antibodies in wild birds from southern Spain. *Epidemiol Infect* **2016**, *144*, 1907-1911.
66. Aguilera-Sepulveda, P.; Gomez-Martin, B.; Aguero, M.; Jimenez-Clavero, M.A.; Fernandez-Pinero, J. A new cluster of West Nile virus lineage 1 isolated from a northern goshawk in Spain. *Transbound Emerg Dis* **2022**, *69*, 3121-3127.
67. Alba, A.; Allepuz, A.; Napp, S.; Soler, M.; Selga, I.; Aranda, C.; Casal, J.; Pages, N.; Hayes, E.B.; Busquets, N. Ecological surveillance for West Nile in Catalonia (Spain), learning from a five-year period of follow-up. *Zoonoses Public Hlth* **2014**, *61*, 181-191.
68. Cano-Terriza, D.; Guerra, R.; Lecollinet, S.; Cerda-Cuellar, M.; Cabezon, O.; Almeria, S.; Garcia-Bocanegra, I. Epidemiological survey of zoonotic pathogens in feral pigeons (*Columba livia* var. *domestica*) and sympatric zoo species in Southern Spain. *Comp. Immunol. Microbiol. Infect. Dis.* **2015**, *43*, 22-27.
69. Figuerola, J.; Angel Jiménez-Clavero, M.; Rojo, G.; Gómez-Tejedor, C.; Soriguer, R. Prevalence of West Nile virus neutralizing antibodies in colonial aquatic birds in southern Spain. *Avian* Pathol **2007**, *36*, 209-212.
70. Gangoso, L.; Grande, J.M.; Llorente, F.; Jiménez-Clavero, M.Á.; Pérez, J.M.; Figuerola, J. Prevalence of neutralizing antibodies to West Nile virus in Eleonora's Falcons in the Canary Islands. *J Wildlife Dis* **2010**, *46*, 1321-1324.
71. Garcia-Bocanegra, I.; Busquets, N.; Napp, S.; Alba, A.; Zorrilla, I.; Villalba, R.; Arenas, A. Serosurvey of West Nile Virus and Other Flaviviruses of the Japanese Encephalitis Antigenic Complex in Birds from Andalusia, Southern Spain. *Vector-Borne Zoonot Dis* **2011**, *11*, 1107-1113.
72. Garcia-Bocanegra, I.; Franco, J.J.; Leon, C.I.; Barbero-Moyano, J.; Garcia-Mina, M.V.; Fernandez-Molera, V.; Gomez, M.B.; Cano-Terriza, D.; Gonzalvez, M. High exposure of West Nile virus in equid and wild bird populations in Spain following the epidemic outbreak in 2020. *Transbound Emerg Dis* **2022**, *69*, 3624-3636.
73. Lopez, G.; Jimenez-Clavero, A.; Tejedor, C.G.; Soriguer, R.; Figuerola, J. Prevalence of West Nile virus neutralizing antibodies in Spain is related to the behavior of migratory birds. *Vector-Borne Zoonotic Dis* **2008**, *8*, 615-621.
74. Martínez-de la Puente, J.; Ferraguti, M.; Ruiz, S.; Roiz, D.; Llorente, F.; Pérez-Ramírez, E.; Jiménez-Clavero, M.Á.; Soriguer, R.; Figuerola, J. Mosquito community influences West Nile virus seroprevalence in wild birds: implications for the risk of spillover into human populations. *Sci Rep* **2018**, *8*, Article 2599.
75. Napp, S.; Montalvo, T.; Pinol-Baena, C.; Gomez-Martin, M.B.; Nicolas-Francisco, O.; Soler, M.; Busquets, N. Usefulness of Eurasian magpies (*Pica pica*) for West Nile virus surveillance in non-endemic and endemic situations. *Viruses* **2019**, *11*, Article 2404.
